# Integrated Comparative Genomic Analysis and Phenotypic Profiling of *Pseudomonas aeruginosa* Isolates From Crude Oil

**DOI:** 10.3389/fmicb.2020.00519

**Published:** 2020-03-31

**Authors:** Anming Xu, Di Wang, Yichen Ding, Yaqian Zheng, Bo Wang, Qing Wei, Shiwei Wang, Liang Yang, Luyan Z. Ma

**Affiliations:** ^1^State Key Laboratory of Microbial Resources, Institute of Microbiology, Chinese Academy of Sciences, Beijing, China; ^2^University of Chinese Academy of Sciences, Beijing, China; ^3^Singapore Centre for Environmental Life Sciences Engineering, Nanyang Technological University, Jurong West, Singapore; ^4^School of Medicine, Southern University of Science and Technology, Shenzhen, China

**Keywords:** *Pseudomonas aeruginosa*, crude oil, alkane hydroxylase, rhamnolipids, *N*-acyl-homoserine lactones

## Abstract

*Pseudomonas aeruginosa* is an environmental microorganism that can thrive in diverse ecological niches including plants, animals, water, soil, and crude oil. It also one of the microorganism widely used in tertiary recovery of crude oil and bioremediation. However, the genomic information regarding the mechanisms of survival and adapation of this bacterium in crude oil is still limited. In this study, three *Pseudomonads* strains (named as IMP66, IMP67, and IMP68) isolated from crude oil were taken for whole-genome sequencing by using a hybridized PacBio and Illumina approach. The phylogeny analysis showed that the three strains were all *P. aeruginosa* species and clustered in clade 1, the group with PAO1 as a representitive. Subsequent comparative genomic analysis revealed a high degree of individual genomic plasticity, with a probable alkane degradation genomic island, one type I-F CRISPR-Cas system and several prophages integrated into their genomes. Nine genes encoding alkane hydroxylases (AHs) homologs were found in each strain, which might enable these strains to degrade alkane in crude oil. *P. aeruginosa* can produce rhamnolipids (RLs) biosurfactant to emulsify oil, which enables their survival in crude oil enviroments. Our previous report showed that IMP67 and IMP68 were high RLs producers, while IMP66 produced little RLs. Genomic analysis suggested that their RLs yield was not likely due to differences at genetic level. We then further analyzed the quorum sensing (QS) signal molecules that regulate RLs synthesis. IMP67 and IMP68 produced more N-acyl-homoserine lactones (AHLs) signal molecules than that of PAO1 and IMP66, which could explain their high RLs yield. This study provides evidence for adaptation of *P. aeruginosa* in crude oil and proposes the potential application of IMP67 and IMP68 in microbial-enhanced oil recovery and bioremediation.

## Introduction

Microorganisms can survive in many harsh environments, such as glaciers, hot springs, and crude oil. In order to survive in these harsh environments, bacteria usually evolve a series of corresponding functional proteins or metabolic systems through adaptive evolution including gene mutation and horizontal gene transfer (HGT) (Ollivier and Magot, 2005). Crude oil is a complex mixture of thousands of compounds that is difficult to be utilized and even toxic for most of bacteria (Xu et al., 2018). Interestingly, bacteria such as *Pseudomonads* can survive in such harsh environment. Alkanes are saturated hydrocarbons of different carbon-chain length and structure, which are the major components of crude oil. Although they are chemically inert, most of them can be efficiently degraded by several microorganisms (Rojo, 2009; Sun et al., 2018). It has been reported that more than 60 genera of aerobic bacteria and 5 genera of anaerobic bacteria are able to degrade n-alkanes (Prince, 2005).

Alkane hydroxylases (AHs), which catalyze the first step of alkane biodegradation, are the key enzymes in aerobic degradation of alkanes by bacteria. These enzymes hydroxylate alkanes to alcohols, which are further oxidized to fatty acids and catabolized via the bacterial β-oxidation pathway (Rojo, 2009). The constituent proteins of the AH system include alkane hydroxylases, one or two rubredoxin (*rubA*, *rubA1*, and *rubA2*) and rubredoxin reductase (*rubB*) (Geissdorfer et al., 1999). Two common AHs in bacteria are the alkane hydroxylase (AlkB)-related AHs and the cytochrome P450 family proteins (Nie et al., 2014a). Furthermore, flavin-binding LC-alkane hydroxylase (*almA*) and thermophilic soluble LC-alkane hydroxylase (*ladA*) have also been found to be involved in the hydroxylation of alkanes (Wentzel et al., 2007; Wang and Shao, 2012). A number of bacteria have been found to contain multiple AHs (van Beilen et al., 2006). For example, the co-existence of AlkB and CYP153 was found in *Dietzia sp.* DQ12-45-1b (Wang et al., 2011; Nie et al., 2014b), and multiple AHs were found in *Amycolicicoccus subflavus* DQS3-9A1^T^ (Nie et al., 2013). Several *Pseudomonas* species have also been proved to have the ability to utilize n-alkane (Kok et al., 1989; Yuste et al., 2000; Zhang et al., 2011). Among them, *P. putida* GPo1 has been extensively studied, which can grow on intermediate chain length n-alkanes by virtue of the OCT plasmid-encoded alkane hydroxylase (Chakrabarty et al., 1973). The OCT plasmid carries the *alk* regulon and consists of two gene clusters: *alkBFGHJKL* and *alkST* (van Beilen et al., 1994, 2001). In recent years, many alkane-degrading *P. aeruginosa* strains have been isolated from crude oil, including *P. aeruginosa* N002, *P. aeruginosa* SJTD-1, and *P. aeruginosa* DQ8 (Zhang et al., 2011; Liu et al., 2012; Das et al., 2015). Genome sequencing has shown that many of these strains contain homologs of the GPo1 alkane hydroxylases genes, which are located on chromosome in contrast to the plasmid-encoded GPo1 (van Beilen et al., 2001).

Under specific environmental conditions, *P. aeruginosa* could produce rhamnolipids (RLs), which are a class of glycolipids biosurfactants and mainly composed of 3-(3-hydroxyalkanoyloxy) alkanoic acids (HAA), mono-rhamnolipids (MRLs) and di-rhamnolipids (DRLs) (Deziel et al., 2003; Soberon-Chavez et al., 2005). In crude oil environment, RLs emulsify crude oil; which is always considered to have prominent ability to solubilize various hydrocarbons for the biodegradation (Beal and Betts, 2000; Noordman and Janssen, 2002). In *P. aeruginosa*, the biosynthesis of RLs is positively regulated by RhlI/RhlR quorum sensing (QS) system that directly activates the transcription of *rhlAB*, which encodes the enzymes catalyzing the first step of RLs biosynthesis. QS is a cell density-dependent communication mechanism which enables bacteria to coordinate cooperative behaviors in response to the accumulation of self-produced autoinducer signals (*N*-acyl-homoserine lactones, AHLs) in their local environment (Passador et al., 1993; Davies et al., 1998; Daniels et al., 2004). In *P. aeruginosa*, QS depends on two AHLs regulatory circuits, Las and Rhl. In the Las system, LasI directs the synthesis of *N*-(3-oxododecanoyl)-homoserine lactone (3-oxo-C12-HSL), which interacts with the transcriptional regulator LasR to control target genes, such as *rhlI/rhlR*. RhlI is also a signal synthase which generates another signal molecular, *N*-butanoyl-homoserine lactone (C4-HSL). Subsequently, C4-HSL combines the signal receptor RhlR to form a RhlR/C4-HSL complex that activates the transcription of several genes, including the *rhlAB* operon (Pearson et al., 1997).

In our previous work, we reported the identification of three rhamnolipid-producing *Pseudomonas* isolates from crude oil (named as IMP66, IMP67, and IMP68), which exhibited sigificant different phenotypes in RLs and pyocyanin production (Das et al., 2014). The 16s rRNA analysis has suggested that two of them (IMP67 and IMP68) are *P. aeruginosa* and IMP66 is identified as *Pseudomonas* sp. Pyr41 (Das et al., 2014). In this study, we performed the comparative genomic analysis for these three *Pseudomonas* strains to further understand their genetic background. Our analysis indicated that all three strains belong to *P. aeruginosa* species. Subsequent analysis showed that these three isolates exhibited a high degree of individual genome plasticity along with abundant numbers of alkane hydroxylase encoding genes, conferring survival advantages for *P. aeruginosa* strains living in the crude oil environment. Moreover, the strains with high yield of RLs produce large amounts of QS signal molecules. This study illustrates the environmental adaptability of *P. aeruginosa*, and proposes potential applications of these strains in bioremediation as well as in microbial-enhanced oil recovery.

## Materials and Methods

### Bacterial Strains, Plasmids, and Culture Conditions

IMP66, IMP67, and IMP68 were isolated from the crude oil of Karamay W#8805, Xin-Jiang province, China. Unless otherwise indicated, *P. aeruginosa* strains were grown in LB without sodium chloride (LBNS) or basal salt medium (BSM, K_2_HPO_4_ 3.815 g, KH_2_PO_4_ 0.5 g, (NH4)_2_HPO_4_ 0.825g, KNO_3_ 1.2625 g, Na_2_SO_4_ 0.2 g, CaCl_2_ 0.02 g, FeCl_3_ 0.002 g, MgCl_2_ 0.02 g/l) at 37°C. Culture was supplemented with 300 μg/ml carbenicillin to select and maintain plasmid-carrying *P. aeruginosa*. A plasmid-borne fusion of the QS-controlled *rhlAB* promoter sequence (423 bp) and GFP coding sequence was used to assess the promoter activity of *rhlAB* in this study. Primers P*_*rhlAB*_*_F (5′-cgccagagcgtttcgac-3′) and P*_*rhlAB*_*_R (5′-ttcacacctcccaaaaattttcgaac-3′) were used to generate an amplicon of the *rhlAB* promoter by I-5^TM^ High-Fidelity DNA Polymerase (Tsingke Biological Technology). The PCR product was purified and ligated into pPROBE-AT vector (Miller et al., 2000) through Gibson assembly (Gibson et al., 2010). Recombinant plasmid pTH22 (*rhlAB*:*gfp*) was then transformed into *Escherichia coli* DH5α and subsequently into *P. aeruginosa* for determination of the transcription of *rhlAB*.

### Genome Assembly and Annotation

Genomic DNA was isolated from an overnight broth culture using the Wizard genomic DNA purification kit (Promega, Madison, WI, United States). A spectrophotometer (NanoDrop 2000 UV-Vis; Thermo Scientific, MA, United States) was used to determine the DNA quantity and ensure the DNA quality was suitable for sequencing. Whole-genome sequencing was performed using the long-read PacBio SMRT sequencing (Pacific Bioscience) platform and the Illumina HiSeq sequencer. Genome sequences were *de novo* assembled using HGAP assembly protocol, which is available with the SMRT Analysis packages and accessed through the SMRT Analysis Portal version 2.1. The pilon v1.5 was then used to correct the assembly (Walker et al., 2014). Annotation was performed using the NCBI Prokaryotic Genome Annotation Pipeline (PGAAP)^1^ (Tatusova et al., 2016). The circular genome map of the three strains including all predicted ORFs with COG functional assignments, rRNA, tRNA, G + C content, and GC skew information were generated using CGview (Stothard and Wishart, 2004).

### Phylogenetic Analysis

The evolutionary position of the three strains relative to other pathovars and species of *Pseudomonas* was determined by multi-locus sequence analysis (MLSA) as described previously (Loper et al., 2012). For phylogenetic tree construction, ten conserved housekeeping genes (*acsA-aroE-dnaE-guaA-gyrB-mutL-ppsA-pyrC-recA-rpoB*) from 51 closely related *Pseudomonas* spp. were retrieved from *Pseudomonas* Genome Database (Winsor et al., 2016). Maximum Likelihood analysis was performed for 100 bootstrap replications in RAxML. The final phylogenetic tree was constructed using the iTOLs software (Letunic and Bork, 2016).

In order to detect the strain-level genome content of the three strains, a rapid large-scale prokaryote pan genome analysis by Roary was performed to identify the conserved (core) and non-conserved (accessory) genes (Page et al., 2015), and then created a concatenated core genome for subsequent phylogenetic analysis. 90 closely related *Pseudomonas* spp. retrieved from *Pseudomonas* Genome Database (Winsor et al., 2016) were used for the core-genome estimating by Roary bacterial genome analysis pipeline. A cut-off with 90% identity was used and the core genes were defined as those presented in 99% of isolates to generate a multi-FASTA alignment file. These data were then used as the input to the next iteration. RAxML was then run over the final multi-FASTA alignment to provide a high-quality phylogenetic tree in newick format. We used a generalized time reversible (GTR) model with gamma (Y) heterogeneity across nucleotide sites and 100 bootstrap replicates. The final phylogenetic tree was constructed using the iTOLs software (Letunic and Bork, 2016).

### Comparative Genome Analysis

The MicroScope platform (Vallenet et al., 2016) provided some of the tools used for the comparative genomic analyses, such as the RGP Finder for determination of regions of genomic plasticity (RGPs). RGPs were defined as DNA segments over 5 kbp possibly related to events of horizontal exchanges, and they were identified using a series of constraints such as G + C% deviation, compositional biases as measured by the tools Alien-Hunter (Vernikos and Parkhill, 2006) and SIGI-HMM (Waack et al., 2006), synteny breaks and proximity to tRNAs. Pan-genome analysis including the genes shared by all these four strains was performed by an “all-against-all” BLAST of the protein sequences of the above genomes using the EDGAR 2.3 software framework (Blom et al., 2016). The genes aligned based on amino acid sequences were used for identifying orthologs. For orthology estimation, EDGAR used a generic orthology threshold calculated from the similarity statistics of the compared genomes, and then generated the veen diagram.

### AHLs Extraction and Quantification

Extraction and quantification of AHLs were based on a previously described method (Ortori et al., 2011). Briefly, strains were grown in LBNS media for 24 h, 1 ml of bacterial cells were spun down and supernatants were harvested for AHLs extraction. Acidified ethyl acetate was used for AHLs extraction and the extraction was repeated three times. The organic layers were combined and evaporated under vacuumrotary evaporation. Residues were dissolved in 50 μl methanol and subjected to LC-MS/MS analysis using mass spectrometer (QTRAP 6500 System, AB SCIEX). The calibration curve was prepared from a linear dynamic range (from 0.33 to 33.3 μM) of standard 3-oxo-C12-HSL (Sigma-Aldrich, Germany).

## Results

### Phylogenetic Analysis of Newly Sequenced Strains With the Other *Pseudomonas* spp.

**FIGURE 1:**
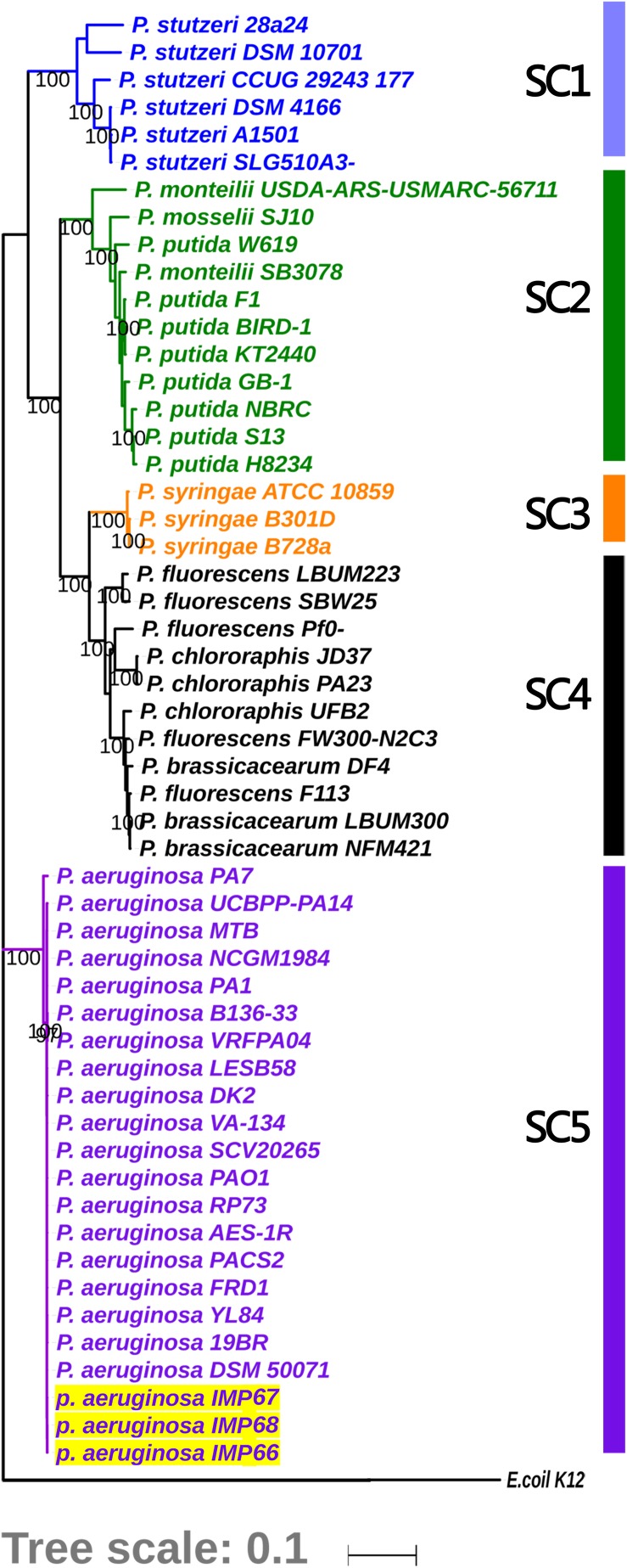
Phylogenetic analysis for depicting the relationships of these three newly sequenced strains with *Pseudomonas* spp. The tree is based on concatenated alignments of ten core housekeeping genes: *acsA, aroE, dnaE, guaA, gyrB, mutL, ppsA, pyrC, recA*, and *rpoB*, and was generated using the RAxML package. Five sequence clusters (SCs) are identified and labeled on the edge of the phylogeny. Clades corresponding to 4 dominant different monophyletic SCs (SC1 to SC3 and SC5) are shown in different colors, while clade for all the strains in the polyphyletic clade (SC4) comprising of the “unclustered” sequences is colored in black. Bootstrap supporting over 90% are labeled for the major nodes. Strains sequenced in this study are shown by yellow shading, which fall into SC5 with *P. aeruginosa*. The *Escherichia coli* K-12 genome was used as an out-group control.

IMP66, IMP67, and IMP68 were isolated from the crude oil of Karamay W#8805, Xin-Jiang province, China. The complete genomes of these three strains were sequenced and assembled. A previous report suggested that only IMP67, and IMP68 were *P. aeruginosa* based on 16S rRNA analysis. It has been showed that the phylogenetic tree based on 16S rRNA lacked resolution at intrageneric level (Anzai et al., 2000). We then used the ten house-keeping genes to further analysis the phylogenetic position of these three strains. A phylogenetic tree was plotted using a maximum likelihood (ML) approach based on multi-locus sequence analysis of 54 representative *Pseudomonas* spp. strains, including 51 stains with complete genome sequence and these three newly sequenced strains (IMP66, IMP67, and IMP68). *E. coli* K12 was used as an out-group. Four tightly monophyletic groups could be identified from the phylogenetic tree based on the 10 concatenated housekeeping genes (Figure 1, SC1 to SC3, and SC5). Strains IMP66, IMP67, and IMP68 were all clustered together within the group of *P. aeruginosa* and were all identified as *P. aeruginosa* strains (Figure 1).

**FIGURE 2:**
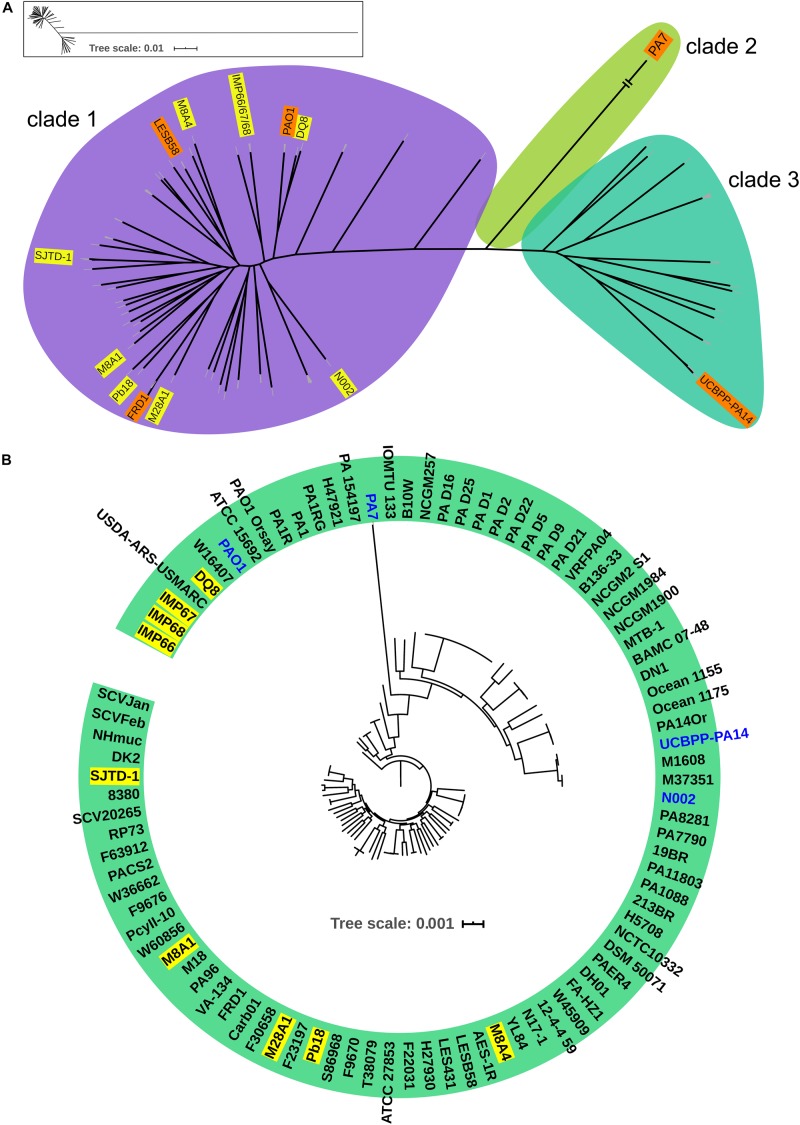
Phylogeny of *P. aeruginosa* population structure. Unrooted **(A)** and circular **(B)** maximum likelihood trees of three newly sequenced crude oil isolates combined with 87 representive *P. aeruginosa* isolates from diverse niches are generated from multiple-sequence alignment with 51,389 SNPs from 820 universally conserved (core) genes. The phylogenetic trees are constructed using RAxML with GTR + γ nucleotide substitution model and 100 bootstrap replicates. Strains are divided into three major clades. Clade 2 is contracted for visualization purpose. A framed miniature of the true appearance of this tree is presented. Representive crude oil sourced *P. aeruginosa* strains are highlighted in yellow.

In order to investigate the evolutionary history of these three newly sequenced strains, we reconstructed the phylogeny of the genus using concatenated core genomes from 90 *P. aeruginosa* isolates, including representative *P. aeruginosa* strains with completely genomes (such as PAO1, PA14, and PA7), IMP66, IMAP67, and IMP68, as well as several crude oil-isolated *P. aeruginosa* strains (Pb18, M8A1, M8A4, M28A1, SJTD-1, DQ8). There were 51,389 SNPs identified in the 820 core genes of the 90 isolates. *P. aeruginosa* strains were divided into three major clades (Figure 2A), which was consistent with the previous reports (Freschi et al., 2015; Castañeda-Montes et al., 2018). Interestingly, all crude oil isolates were consistently distributed in clade 1, which was represented by strain PAO1 and contained the strains from a wide range of sources, including natural environment and clinical settings (Figure 2A). Although most of the strains from crude oil were randomly distributed within clade 1, strain IMP66, IMP67, and IMP68 were clustered together (Figure 2B), which was consistent with the results of housekeeping gene analysis (Figure 1). Taken together, our data suggested that these three *P. aeruginosa* strains were closely related in the evolutionary process.

### Comparative Genomic Analysis of IMP66, IMP67, IMP68, and PAO1

**FIGURE 3:**
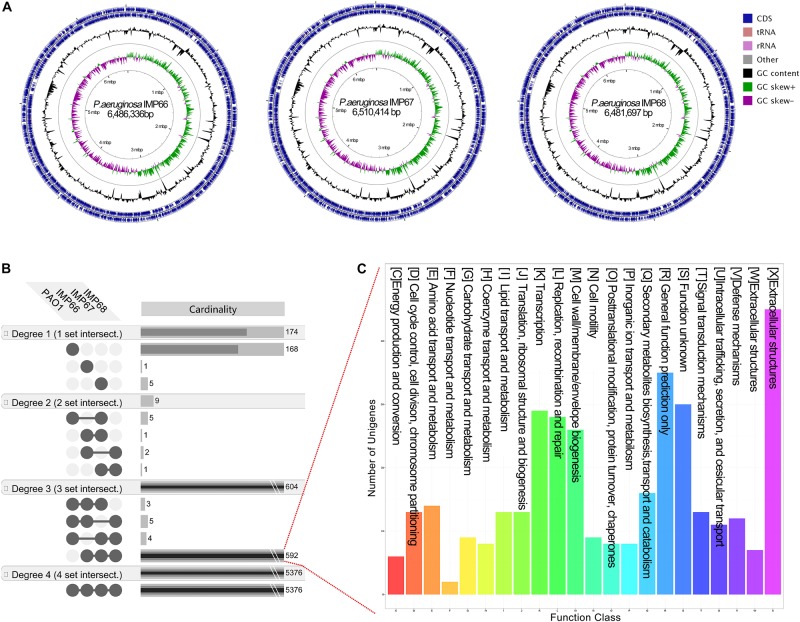
**(A)** Circular chromosome map of *P. aeruginosa* IMP66, IMP67, and IMP68. Pan-genome analysis of three crude-oil sourced *P. aeruginosa* isolates and PAO1. **(B)** The unique and shared genes among *P. aeruginosa* IMP66, IMP67, IMP68 and PAO1 are performed using the EDGAR software platform based on the orthology analysis. The results are visualized in four degrees. Degree one represents the distinct singletons harbored by each single strain; degree two represents the genes shared by every two strains; degree three represents the genes shared by every three strains; degree four is the number of core genes shared by all the four strains. **(C)** COG annotation of all 592 exclusive CDSs shared by these three *P. aeruginosa* isolates.

IMP66, IMP67, and IMP68 were similar in genome size, with a single chromosome of about 6.5 Mb (Table 1 and Figure 3A). The three strains had the same G + C% content of 66.4% (Table 1), which was consistent with other *P. aeruginosa* strains. The total numbers of coding sequences (CDSs) in the three strains were close to 6100, which were much higher than that of PAO1. We also performed a general comparison of the genomic features including genome size, GC content, and predicted number of CDSs among several crude oil sourced strains and well-studied strain PAO1 (Table 1). The results showed that most of the crude oil isolates harbored bigger genome size than PAO1 strain except SJTD-1. However, SJTD-1 possessed about 200 more CDSs than PAO1 even with similar genome size. These results suggested that genome expansions might confer survival advantages for these isolates living in crude oil.

**TABLE 1:** General genome properties comparison of the completely sequenced crude oil isolates with laboratory strains.

Genome features*	**Values**								
	**PAO1**	**IMP66**	**IMP67**	**IMP68**	**Pb18**	**M8A1**	**M8A4**	**M28A1**	**SJTD-1**
Size (bp)	6,264,404	6,486,336	6,500,677	6,481,180	6,401,521	6,368,297	6,349,902	6,499,441	6,243,825
CDS (total)	5697	6077	6096	6070	5,823	5,803	5,766	5,913	5,808
CDS (coding)	5572	5996	6007	5991	5,684	5,687	5,671	5,772	5,761
G + C (%)	66.6	66.4	66.4	66.4	66.2	66.4	66.4	66.1	66.5

*The features identified for each category were retrieved from NCBI after submission and annotation of genome data.

**FIGURE 4:**
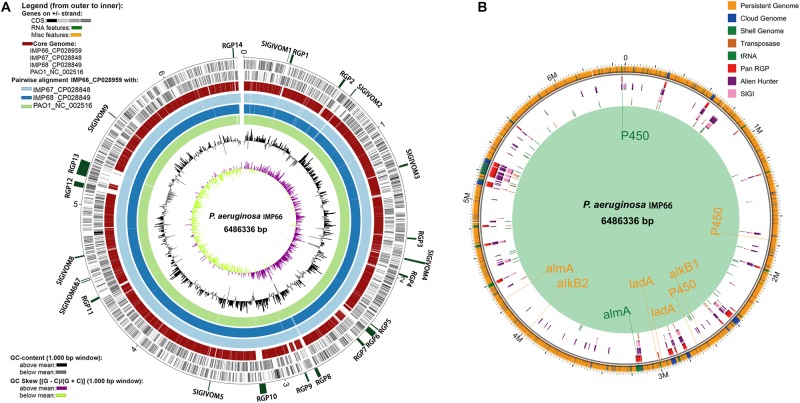
Whole genome comparison of IMP66, IMP67, IMP68, and PAO1. **(A)** Analysis of regions of genomic plasticity among IMP66, IMP67 and IMP68 (IMP66 was used as a representative of the three strains). The innermost ring represents the G + C skew of IMP66, followed by its G + C content. Light green, dodger blue and light blue rings that follow depict BLASTN comparisons between the genome of IMP66 and those of strains PAO1, IMP67, and IMP68, respectively. The red ring represents the core genome of the four strains. Then the two black rings are the CDSs of IMP66. The outermost interspaced ring (in dark green) represents the localization of the predicted regions of genomic plasticity (RGP) in the IMP66 genome, and the labels of each region follow the ones in Table 2. **(B)** Regions with different colors represent potentially horizontally transferred genes (HGT) which are gathered in genomic regions (Region of Genomic Plasticity, RGP). All potential alkane hydroxylases were marked at the chromosome with their corresponding colors.

We then analyzed the group of shared and unique genes between IMP66, IMP67, IMP68, and PAO1. All the shared and unique genes were determined and visualized by EDGAR platform. The four bacteria shared a conserved core genome comprising of 5,376 CDSs. PAO1 possessed 168 distinct singletons, which was the largest number of singletons among these four strains. Interestingly, there were 592 unique genes shared by IMP66, IMP67, and IMP68 (Figure 3B). The largest portion (46 genes) of the 592 unique genes belonged to mobilomes (prophages and transposons, category X) according to the COG annotation. Other major unique genes were in category K (transcription), category L (replication, recombination, and repair), and category M (cell wall/membrane/envelope biogenesis), respectively. A small portion of genes were related to energy production and conversion, amino acid transport, and metabolism, etc (Figure 3C). The mobilome genes are commonly associated with HGT as they often carry transposons, phage fragments, and IS elements. These results suggested that HGT might commonly occur in these three strains. To further investigate HGT events in these three strains, the MicroScope platform was used to compare the genomes of IMP66, IMP67, IMP68 with PAO1. We selected IMP66 genome as the representative reference (the same results were obtained when using IMP67 or IMP68) of these three strains against the query genome of strain PAO1. A total of 23 conserved RGPs were identified in IMP66 (Figure 4A and Table 2). Most of these regions contained the sequences related to transposable elements and were flanked by genes coding for tRNAs or phage-structures (Table 2), indicating the possible horizontal gene acquisition. Although most of these regions encoded proteins of unknown functions, some RGPs with important genomic information were identified. RGP9 carried an insertion sequence at its 5′-end (IS3 family, DBX28_13665) and harbored CDSs with predicted functions that belonging to type I-F CRISPR-Cas system. RGP10 is a 48.9 kb genomic element with 56 CDSs including the genes for IS3 family transposases and a possible alkane degradation cluster with a long-chain alkane hydroxylase (*almA*, DBX28_14785). A noteworthy plasticity region with an aromatic ring-hydroxylating dioxygenase (DBX28_20755) was found in RGP11 (Table 2), which might provide competitive advantages for these three strains living in crude-oil areas. A large number of RGPs detected in IMP66, IMP67, and IMP68 indicated a complex exchange pattern of genetic material segments that occurred during the chromosome reshaping process throughout the evolutionary history.

**TABLE 2:** Regions of Genomic Plasticity (RGPs) identified in the chromosome of IMP66, IMP67, and IMP68.

**Region**	**Coordinates**	**Length (kbp)**	**No. of CDSs**	**G + C%**	**Features**	Best hit (% coverage,% identity)*
RGP1	290261–302956	12696	22	57.9	tRNA	*P. aeruginosa* T63266 (100%, 100%)
RGP2	631096–652201	21106	35	57.8	tRNA, transposase, integrase	*P. aeruginosa* PAER4_119 (44%, 94.1%)
RGP3	1730348–1738782	8435	15	56	tRNA	*P. aeruginosa* T63266 (100%, 99.9%)
RGP4	1975456–1982437	6982	14	49.9	unknown function	*P. aeruginosa* PAK (100%, 99.9%)
RGP5	2339727–2346716	6990	22	65.5	tRNA	*P. aeruginosa* T63266 (100%, 100%)
RGP6	2366073–2406282	40210	64	63.5	integrase, phage	*P. aeruginosa* F63912 (88%, 97.4%)
RGP7	2461950–2475724	13775	18	57.3	tRNA, transposase	*P. aeruginosa* T63266 (100%, 100%)
RGP8	2798834–2807221	8388	28	49.5	Transposase	*P. aeruginosa* T63266 (100%, 99.9%)
RGP9	2858761–2870683	11923	20	62.5	transposase,type I-F CRISPR	*P. aeruginosa* PABL017 (100%, 94.6%)
RGP10	3122707–3171699	48993	56	61.9	transposase, ***alkane hydrolase***	*P. aeruginosa* T63266 (100%, 100%)
RGP11	4391909–4404142	12234	21	61.8	***Aromatic ring-hydroxylating dioxygenase*** subunit alpha, acetyltransferase	*P. aeruginosa* T63266 (100%, 100%)
RGP12	5104668–5141335	36668	65	64.5	transposase, phage	*P. aeruginosa* JD024 (90%, 97.3%)
RGP13	5178764–5291282	112519	148	60.8	tRNA, transposase, type II toxin-antitoxin system, integrating conjugative element protein, methyl-accepting chemotaxis protein	*P. aeruginosa* B136-33 (69%, 97.4%)
RGP14	6456744–6463635	6892	15	60	***cytochrome P450***, isopenicillin N synthase family oxygenase	*P. aeruginosa* T63266 (100%, 100%)
SIGIVOM1	280000–283595	3596	11	60.4	transposase	*P. aeruginosa* T63266 (100%, 100%)
SIGIVOM2	731236–769656	38421	62	58.9	tRNA	*P. aeruginosa* SJTD-1 (100%, 99.8%)
SIGIVOM3	1287864–1292500	4637	13	58.2	unknown function	*P. aeruginosa* M8A1 (100%, 99.7%)
SIGIVOM4	1869178–1874512	5335	16	47.3	diguanylate phosphodiesterase	*P. aeruginosa* T63266 (100%, 100%)
SIGIVOM5	3479225–3484687	5463	14	45.5	unknown function	*P. aeruginosa* T63266 (100%, 100%)
SIGIVOM6	4501618–4515000	13383	23	59.6	tRNA, type 1 fimbrial protein	*P. aeruginosa* M8A1 (100%, 99.8%)
SIGIVOM7	4522500–4525211	2712	13	63.1	unknow function	*P. aeruginosa* T63266 (100%, 100%)
SIGIVOM8	4669092–4686046	16955	39	57.9	integrase, phage	*P. aeruginosa* T63266 (77%, 100%)
SIGIVOM9	5590764–5595000	4237	19	57.1	phage	*P. aeruginosa* UCBPP-PA14 (67%, 97.8%)

*BLASTN performed against the “nr” database at the NCBI website.

### Analysis of Genes Involved in Alkane Degradation

Alkane hydroxylases (AHs) catalyzes the first step of alkane degradation and is involved in an AH system that includes electron transport protein rubredoxin and rubredoxin reductase. In the present study, whole-genome screening was used for identification of *alkB*, P450, *almA*, and *ladA* from IMP66, IMP67, and IMP68 through blasting against the reported AHs. We observed that the three strains carried up to 9 AH-encoding genes including two *alkB* homologous genes, three P450 homologous genes, two *almA* homologous genes and two *ladA* homologous genes (Figure 4B). As the “persistent genome” is generally considered to be more stable replicons, it is not surprising that most of the alkane hydroxylase genes were located in the “persistent genome”. Interestingly, two alkane hydroxylases were found in the “shell genome” (an *almA* gene in RGP10, and a cytochrome P450 gene in RGP14) (Figure 4B and Table 2). RGP10 was later identified to be a possible alkane degradation cluster that contained the genes coding for alkane hydroxylase (*almA*, DBX28_14785), SDR family oxidoreductase (DBX28_14685, DBX28_14840), phytanoyl-CoA dioxygenase (DBX28_14740, DBX28_14775), NAD(P)/FAD-dependent oxidoreductase (DBX28_14785, DBX28_14815), and NAD-dependent alcohol dehydrogenase (DBX28_14795). Almost all the genes in RGP10 were related to metabolism and energy production (Table 3). These results suggested that these three strains might have the ability to degrade and utilize a broad-spectrum of alkanes for growth.

**TABLE 3:** Detail information of the probable alkane degradation cluster in RGP10.

**Label**	**Begin**	**End**	**Type**	**Product**	**GC Region**	**SIGI**	**IVOM**
DBX28_14665	3119086	3120522	CDS	undecaprenyl-phosphate glucose phosphotransferase	−	−	−
DBX28_14670	3120517	3120645	CDS	Glycosyltransferase	−2SD	−	−
DBX28_14675	3121054	3121725	CDS	hypothetical protein	−	−	−
DBX28_14680	3121772	3122491	CDS	MOSC domain-containing protein	+1SD	−	−
DBX28_14685	3122707	3123447	CDS	SDR family oxidoreductase	−	−	+
DBX28_14690	3123497	3123859	CDS	hypothetical protein	−	+	+
DBX28_14695	3124233	3125303	CDS	Hydrolase	−	+	+
DBX28_14700	3125694	3129635	CDS	hypothetical protein	−2SD	+	+
DBX28_14705	3129746	3130042	fCDS	IS3 family transposase	−1SD	+	+
DBX28_14705	3130043	3130573	fCDS	IS3 family transposase	−	+	+
DBX28_14705	3130600	3130725	fCDS	IS3 family transposase	−	+	+
DBX28AM_2945	3130645	3130875	CDS	protein of unknown function	−2SD	+	+
DBX28_14710	3130846	3131091	CDS	hypothetical protein	−2SD	+	+
DBX28_14715	3131276	3131785	CDS	hypothetical protein	−1SD	+	+
DBX28_14720	3132014	3132589	CDS	hypothetical protein	−2SD	+	+
DBX28_14725	3132719	3132985	fCDS	IS3 family transposase	−1SD	+	+
DBX28_14725	3132988	3133407	fCDS	IS3 family transposase	−	+	+
DBX28_14725	3133431	3133694	fCDS	IS3 family transposase	−2SD	+	+
DBX28AM_2951	3133851	3133934	CDS	protein of unknown function	−2SD	+	+
DBX28AM_2952	3133941	3134135	CDS	protein of unknown function	−1SD	+	+
DBX28_14730	3134510	3135052	CDS	hypothetical protein	−2SD	+	+
DBX28_14735	3135075	3136274	CDS	hypothetical protein	−	+	+
DBX28_14740	3136568	3137434	CDS	phytanoyl-CoA dioxygenase family protein	−1SD	+	+
DBX28_14745	3137519	3138481	CDS	glycosyl hydrolase	−	+	−
DBX28_14750	3138492	3140894	CDS	RND transporter	−	−	+
DBX28_14755	3140903	3142489	CDS	DUF1302 domain-containing protein	−	−	+
DBX28_14760	3142573	3143997	CDS	DUF1329 domain-containing protein	−	−	+
DBX28AM_2960	3144149	3144388	CDS	protein of unknown function	−2SD	+	+
DBX28_14765	3144552	3145199	CDS	hypothetical protein	−2SD	+	+
DBX28_14770	3145310	3146677	CDS	FAD-binding oxidoreductase	−	+	+
DBX28_14775	3146739	3147626	CDS	phytanoyl-CoA dioxygenase family protein	−1SD	+	+
DBX28_14780	3148075	3148656	CDS	TetR/AcrR family transcriptional regulator	−1SD	+	+
DBX28_14785	3149040	3150566	CDS	NAD(P)/FAD-dependent oxidoreductase, almA like	−1SD	+	+
DBX28_14790	3150563	3151438	CDS	alpha/beta hydrolase	−	+	+
DBX28_14795	3151669	3152826	CDS	NAD−dependent alcohol dehydrogenase	−	−	+
DBX28_14800	3152839	3154170	CDS	APC family permease	−	−	−
DBX28_14805	3154249	3155673	CDS	gamma-aminobutyraldehyde dehydrogenase	−	−	−
DBX28_14810	3155719	3156972	CDS	aspartate aminotransferase family protein	−	−	−
DBX28_14815	3157060	3157980	CDS	NAD(P)-dependent oxidoreductase	−	−	−
DBX28_14820	3158054	3158206	CDS	amino acid permease	−1SD	−	−
DBX28_14825	3158284	3159942	CDS	cation acetate symporter	−	−	−
DBX28_14830	3159939	3160262	CDS	DUF485 domain-containing protein	−	−	−
DBX28_14835	3160319	3161968	CDS	acyl-CoA synthetase	−	−	−
DBX28_14840	3162052	3162819	CDS	SDR family NAD(P)-dependent oxidoreductase	+ 1SD	−	−
DBX28_14845	3162819	3163991	CDS	acyl-CoA dehydrogenase	−	−	−
DBX28_14850	3164042	3164398	CDS	acyl-CoA dehydrogenase	−	−	−
DBX28_14855	3164395	3165450	CDS	phosphotransferase family protein	−	−	−
DBX28_14860	3165647	3167599	CDS	sigma-54-dependent Fis family transcriptional regulator	−	−	−
DBX28_14865	3167665	3168168	CDS	transcriptional regulator	−1SD	−	−
DBX28_14870	3168227	3170530	CDS	MCE family protein	−	−	−
DBX28_14875	3170523	3171143	CDS	paraquat-inducible protein A	−	−	−
DBX28AM_2983	3171472	3171699	CDS	conserved protein of unknown function	−1SD	−	−
DBX28_14880	3171792	3173375	CDS	aldehyde dehydrogenase [NADP(+)]	+ 1SD	−	−
DBX28_14885	3173504	3174499	CDS	FAH family protein	−	−	−
DBX28_14890	3174527	3175702	CDS	L-rhamnonate dehydratase	−	−	−
DBX28_14895	3175733	3177055	CDS	MFS transporter	−	−	−

### Difference in RLs Production Among the Three Strains is Not at Genomic Level

Our previous study described that three strains produced different amounts of RLs. IMP67 and IMP68 produced much larger amounts of RLs than IMP66 (Das et al., 2014). The pan-genome analysis found that IMP67 and IMP68 possessed sereval unique genes compared to IMP66 (Figure 3B). These unique genes belong to type II secretion system, lipopeptide, non-ribosomal peptide synthetase, and hypothetical protein, which were not reported to be directly relevant to the RLs biosynthesis. Moreover, the average nucleotide identity (ANI) value between IMP66 and IMP68 is 99.99%. These results implied that the reason for the different yield of RLs of these strains is unlikely at genomic level.

**FIGURE 5:**
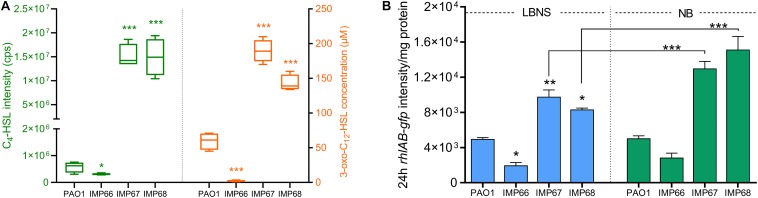
*rhlAB* transcription and AHLs quantification. **(A)** The concentration of C4-HSL (right panel) and 3-oxo-C12-HSL (left panel) of these three newly sequenced strains and laboratory strain PAO1. The concentration of C4-HSL is directly displayed using the original peak area due to no standard C4-HSL. **(B)** The *rhlAB* transcription level of PAO1, IMP66, IMP67 and IMP68 are indicated by the green fluorescent intensity of plasmid P*rhlAB*:*gfp* after 24 h incubation in LBNS or NB broth. Significance determined using Student’s *t*-test, *p < 0.05; **p < 0.01; ***p < 0.001, error bars represent the SD.

To find the reason for the difference in RLs production among three strains, we then detected the QS signal molecules (C4-HSL and C12-HSL) by LC-MS/MS quantification, which positively regulate the expression of rhlAB, the two key RLs synthesis genes. After 24 h incubation, IMP67 and IMP68 produced significantly higher (3–10-folds) level of the two QS signal molecules (C4-HSL and C12-HSL) than that of PAO1, yet IMP66 produced trace amount of C4-HSL as well as C12-HSL, which might explain the reason for the less RLs production in IMP66 (Figure 5A). In addition, we used the *rhlAB*:*gfp* fluorescence-based reporter plasmid pTH22 to track the intracellular *rhlAB* transcription level. GFP fluorescence intensity was normalized by the whole cell protein concentration. IMP67 and IMP68 presented higher *rhlAB* expression than that of IMP66 and PAO1 (Figure 5B), which is correlated with the RLs and QS signal molecule production. Moreover, *rhlAB*-*gfp* intensity was significantly improved when IMP67 and IMP68 were grown in the nutrient broth (NB) medium (Figure 5B), which is a known RLs-promoting medium, suggesting that *rhlAB* transcription is related to the nutrient in the growth medium.

**FIGURE 6:**
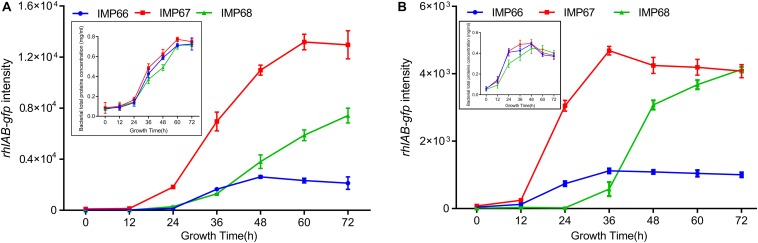
*rhlAB* transcription of IMP66, IMP67, and IMP68 when grown with n-alkane as the sole carbon source. C20 **(A)** or C24 **(B)** was used as the sole carbon source for the growth of IMP66, IMP67, and IMP68 carrying the reporter plasmid P*rhlAB*:*gfp* in BSM broth. The expression level of P*rhlAB*:*gfp* is monitored and recorded as the flourescent intensity of GFP. The growth curve presented by bacterial total protein concentration was shown as the inset.

Rhamnolipids can enhance the bioavailability of crude oil, and therefore are crucial for microbial consortium living in the crude oil environment. To investigate whether these three strains possess the ability to produce RLs when grown with crude oil, especially n-alkanes, as the sole carbon source. We used again the *rhlAB*:*gfp* to determine the transcription of *rhlAB* when they cultured with alkane (tetracosane, C24; eicosane, C20) as the sole carbon source. The three strains displayed noticeable growth indicating by total protein concetration, and *rhlAB* transcription after 72 h incubation in the two types of n-alkanes, while they exhibited similar growth rate and growed better in C20 than C24 (Figures 6A,B). Altogether, our data indicated that high yield of RLs in IMP67 and IMP68 was due to the transcriptional regulation, not at genomic level.

## Discussion

The environmental microorganism *P. aeruginosa* possesses great ecological flexibility, which enables it to thrive in diverse ecological niches. Under different environmental settings, *P. aeruginosa* may use different strategies to survive, such as forming biofilms in high-temperature oilfields (Orphan et al., 2000). It was widely believed that crude oil is a harsh habitat for microbes because of its high toxicity and hydrophobicity. However, growing evidence has revealed the presence of living microbes in crude oil (Yoshida et al., 2005). It has been reported that microbial communities in the crude oil phase were dominated by *Pseudomonas*, accounting for 96.84–98.87% of the total OTUs (Yamane et al., 2008; Cai et al., 2015), which exhibited significant survival advantages over other genera. In this study, we reported that three *P. aeruginosa* strains isolated from crude oil, IMP66, IMP67, and IMP68, exhibited excellent capabilities in n-alkane utilization and RLs production, which are two important factors for *P. aeruginosa* strains to maintain sustainable growth in the crude oil environment. Our work provides a better understanding of the adaptability of *P. aeruginosa* in crude oil.

Alkanes are the main components of petroleum hydrocarbons, and therefore, alkane degradation ability is critical for microorganisms to survive in petroleum-contaminated environments. Alkane hydroxylases are the key enzymes for aerobic degradation bacteria. Previous reports have identified AHs in several *P. aeruginosa* strains cultured from petroleum-contaminated environments (Zhang et al., 2011; Liu et al., 2012; Das et al., 2015). Compared to these *P. aeruginosa* strains, IMP66, IMP67, and IMP68 possess more AHs related genes, especially the coding genes for long-chain alkane monooxygenase, *almA*, and *ladA*. The coexistence of multiple alkane hydroxylases including *alkB*, P450, *almA*, and *ladA* in one bacterium is quite common in *Marinobacter* strains (Sun et al., 2018), *Dietziasp.* DQ12-45-1b (Nie et al., 2011), *Amycolicicoccus subflavus* DQS3-9A1T(AEF42720) (Nie et al., 2013), *Acinetobacter* spp. ADP1 (Barbe et al., 2004), and was also observed in many *Alcanivorax* isolates (Wang et al., 2010; Wang and Shao, 2012). However, nine AH genes identified in one single chromosome is rare in *P. aeruginosa* species. These alkane hydroxylases were demonstrated to be complementary and cover expanded substrate ranges, thereby enhancing the adaptive ability of *P. aeruginosa* in crude oil.

Gene duplication and horizontal gene transfer are common evolutionary processes that generate novel genes or functions for rapid adaptation (Kondrashov et al., 2002; Gogarten and Townsend, 2005). In this study, *alkB*, P450, *almA* and *ladA* paralogs with multiple copies were found in IMP66, IMP67, and IMP68. Moreover, a P450 and an *almA* gene were found integrated into the chromosome presumably through HGT (Figure 3B). These paralogs may exhibit different functions. For example, *alkW1* and *alkW2* are two paralogous genes encoding AlkB rubredoxin fusion proteins, which has been reported originally in *Dietzia* sp. DQ12-45-1b. It was shown that AlkW1 could hydroxylate n-alkanes ranging from C14 to C32, whereas AlkW2 was not expressed in this condition (Nie et al., 2011). Results from the whole genome comparison and pan-genome analysis have indicated that many flanking mobile genetic elements exist in the genomes of IMP66, IMP67, and IMP68. For instance, a 50 kb-long alkane metabolism-related genomic island (RGP10) containing an *almA*-like (50% identity) alkane monooxygenase along with several IS3 transposases is found in these three strains (Tables 2, 3), suggesting that horizontal gene transfer may occur during their life cycles.

*Pseudomonas aeruginosa* is previously demonstrated to use QS to regulate the synthesis of RLs, which are critical for *P. aeruginosa* to emulsify hydrocarbons and make them easier for uptake and assimilation. However, RLs also act as costly extracellular public goods. When the population growth was limited by restricted nutritional conditions, the regulation of QS-controlled public-good related genes may exhibit a metabolic prudent manner (Xavier et al., 2011). This could be a reasonable explanation for the fact that IMP67 and IMP68 expressed huge amount of QS signal molecules (Figure 5A), yet produced just a little higher amount of RLs than PAO1 (Das et al., 2014). Another plausible explanation could be that both RLs and exopolysaccharides share the common sugar precursors catalyzed by AlgC (Olvera et al., 1999; Wang et al., 2014). It could be a tactic for *P. aeruginosa* to synthese proper amount of RLs even with excess AHLs, and balance the synthesis of RLs and exopolysaccharides.

In conclusion, our results have showed that the alkane degradation ability and the superior AHL synthesis ability are two promoting factors for *P. aeruginosa* adaption in crude oil environment, which may also confer these three new isolates potential engineering performance in bioremediation of crude-oil pollution as well as microbial-enhanced oil recovery. Moreover, the native type I-F CRISPR-Cas system possessed by IMP66, IMP67, and IMP68 could be further established as a highly efficient, *in situ* genome-editing technique to modify the strains for future applications in such as industrial RLs production.

## Data Availability Statement

The assembled annotated complete genomes used in this study are available at the National Center for Biotechnology Information (NCBI), under the accession code CP028959, CP028848 and CP028849. The other sequenced genomes are abtained from *Pseudomonas* database (www.pseudomonas.com).

## Author Contributions

LM and SW acquired the funding. DW and LM designed the experiments. AX, YD, YZ, BW, and QW carried out the work and made contributions to acquisition of the data. AX wrote the original draft. LM, DW, LY and SW revised and edited the manuscript.

## Conflict of Interest

The authors declare that the research was conducted in the absence of any commercial or financial relationships that could be construed as a potential conflict of interest.

## Footnotes

1 https://www.ncbi.nlm.nih.gov/genome/annotation_prok/
